# Seeing Touches Early in Life

**DOI:** 10.1371/journal.pone.0134549

**Published:** 2015-09-14

**Authors:** Margaret Addabbo, Elena Longhi, Nadia Bolognini, Irene Senna, Paolo Tagliabue, Viola Macchi Cassia, Chiara Turati

**Affiliations:** 1 Department of Psychology, University of Milan-Bicocca, Piazza Ateneo Nuovo 1 (U6), 20126, Milano, Italy; 2 NeuroMi, Milan Center for Neuroscience, Milano, Italy; 3 Research Department of Clinical, Educational and Health Psychology, University College London, London, United Kingdom; 4 Laboratory of Neuropsychology, Istituto Auxologico Italiano, Milano, Italy; 5 Cognitive Neuroscience Department and Cognitive Interaction Technology-Center of Excellence, Bielefeld University, Bielefeld, Germany; 6 Neonatology and Intensive Care Unit, MBBM Foundation, San Gerardo Hospital, 20900, Monza, Italy; University G. d'Annunzio, ITALY

## Abstract

The sense of touch provides fundamental information about the surrounding world, and feedback about our own actions. Although touch is very important during the earliest stages of life, to date no study has investigated infants’ abilities to process visual stimuli implying touch. This study explores the developmental origins of the ability to visually recognize touching gestures involving others. Looking times and orienting responses were measured in a visual preference task, in which participants were simultaneously presented with two videos depicting a touching and a no-touching gesture involving human body parts (face, hand) and/or an object (spoon). In Experiment 1, 2-day-old newborns and 3-month-old infants viewed two videos: in one video a moving hand touched a static face, in the other the moving hand stopped before touching it. Results showed that only 3-month-olds, but not newborns, differentiated the touching from the no-touching gesture, displaying a preference for the former over the latter. To test whether newborns could manifest a preferential visual response when the touched body part is different from the face, in Experiment 2 newborns were presented with touching/no-touching gestures in which a hand or an inanimate object—i.e., a spoon- moved towards a static hand. Newborns were able to discriminate a hand-to-hand touching gesture, but they did not manifest any preference for the object-to-hand touch. The present findings speak in favour of an early ability to visually recognize touching gestures involving the interaction between human body parts.

## Introduction

In our daily interactions with the animate and the inanimate world, we observe touch in many situations and we attribute to it communicative and affective meanings. Among other sensory modalities, touch is the first to develop in utero and represents a primary means of learning and exploring the environment from fetal life throughout infancy [1].

Although it is commonly recognized that touch is very important early in life [2, 3], we know very little about how infants visually perceive others being touched. To our knowledge, so far no study has investigated infants’ ability to visually distinguish between touching and no-touching gestures. Conversely, visual processing of touch has been extensively investigated in adults [4–17].

Recent neuroimaging studies have shown that, when adults witness another person or even an object being touched by a human agent, neural circuitries normally recruited during the self-experience of touch are activated [4, 7–12]. Activation of the somatosensory cortices during touch observation is further supported by brain stimulation [5, 6, 13] and lesion studies [14], the first showing the selectivity of the human somatosensory cortex for the visual processing of contact between human body-parts [13]. This evidence is in line with the view that adult humans are equipped with a “tactile mirror system”, which matches observed touch with the sense of one’s own touch [15]. This system has been proposed to play a key role in social perception and empathy [5, 6, 13, 15–17].

Already during prenatal life, infants acquire extensive somatosensory-motor experience. In the womb fetuses perform different hand movements directed towards the environment, like the uterine wall, the umbilical cord or their own body, in particular the face [18, 19]. These movements appear to be well organized and coordinated [20, 21, 22]. At 24 weeks of gestation, half of the fetal arm movements result in hands touching the mouth [21], with the frequency of touch for lower and perioral regions of the face increasing significantly with gestational age. Hand-to-face gestures represent one of the most frequent activities during fetal life, and they remain a prominent behavior during the earlier stages of postnatal life [23]. In fact, neonates often touch their face, and especially their mouth, during their waking hours [24, 25].

Possibly due to prenatal somatosensory experiences, neonates seem to have some knowledge about their body immediately after birth [26, 27, 28]. For example, they are able to discriminate between external touch and self-stimulation, displaying rooting responses when their cheek is touched by the finger of somebody else, but not by their own finger [27]. Likewise, newborns imitate observed facial and hand movements [29, 30].

With respect to the visual processing of tactile bodily signals, Zmyj and colleagues [31] have shown that 7- and 10-month-old infants are sensitive to intersensory visual-tactile contingency. When presented with two videos displaying a lifelike-baby doll whose legs are touched by a hand, infants look longer at the video in which the touch is contingent with a tactile stimulation on their own leg than at a non-contingent touch. By contrast, when presented with a video showing oblong wooden blocks rather than doll legs, infant do not show any visual preference, even in the case of contingent tactile stimulation. A recent study by Filippetti et al. (2013) further showed that 1-to-4-day old newborns look longer towards a video displaying a paintbrush stroking an infant’s cheek in synchrony with a tactile stimulation on their own cheek, than towards a video in which the stroke was asynchronous. Newborns show such a preference only when the observed face is depicted in its canonical upright orientation, but not when it is inverted [26]. The ability to detect intersensory synchrony is seen as crucial in the development of an early sense of one’s own body. In fact, the early ability to match the rhythm of a corporeal sensory event to a non-corporeal one provides infants with critical information fundamental to perceive their own body as a differentiated object among other objects in the world [32].

Unlike cross-modal visual-tactile capabilities, so far infants’ ability to recognize tactile stimulations on others' body solely based on visual information has not been investigated. Rather, research has focused on infants’ visual perception of goal-directed movements [33, 34, 35, 36] showing that from the earliest stages of postnatal life newborns are able to discriminate between visual cues indicating goal-directed and non-goal-directed actions, and prefer the former to the latter [37]. Although it is clear that young infants detect goal-directed hand gestures, it is still unknown whether and at which stage of development they can visually recognize hand gestures depicting touch.

By touching their own face during prenatal life infants gain a great deal of somatosensory-motor experience, which might bootstrap visual discrimination between touching and no-touching gestures from the earliest stages of postnatal life. We have addressed this issue in two experiments using a visual preference task in which two dynamic images depicting a touching gesture and a no-touching gesture involving a face, a hand or an object were presented; looking times and orienting responses were measured. In Experiment 1, 2-day-old (i.e., newborn) and 3-month-old infants were shown gestures involving a moving hand approaching a static face. In Experiment 2, 2-day-old infants were assigned to two conditions, different with respect to the agent performing the gesture: in one condition newborn infants were presented with gestures involving a hand approaching another hand, while in the other condition the agent that performed the gesture was an object.

## Experiment 1

Here we explored the ability of newborns and 3-month-old infants to discriminate between dynamic hand-to-face touching and no-touching gestures. Participants were tested with an infant-control visual preference paradigm, in which they were simultaneously presented with two dynamic images depicting a hand moving towards a static face. In one video, the hand movement ended up with a hand-to-face contact (i.e., touching gesture), in the other video the hand movement terminated before the hand-to-face contact occurred (i.e., no-touching gesture). If prenatal experience is sufficient for developing the ability to discriminate between gestures with or without a tactile component, newborns would discriminate between the two hand gestures. Discrimination would be implied by a significant preference for either the touching gesture, which might be associated with sensorimotor experiences and rewards (i.e. affective touch), or the no-touching gesture, which might be perceived as an unfamiliar, unexpected event.

### Method

#### Participants

The final sample included 18 healthy full-term newborns (10 girls; mean age: 48 h, range: 26–85, mean birth weight: 3177g, Apgar score: at least 8 after 5 minutes) and 18 3-month-old infants (10 girls; mean age: 94 days, range: 82–103 days). Only one 3-month-old and none of the newborns reported to have a twin.

Newborns were recruited at the maternity unit of the San Gerardo Hospital in Monza while 3-month-old infants were recruited via a database of parents who had agreed to participate in the study. Five additional newborns and six 3-month-olds were tested but excluded from the final sample due to fussiness (*n* = 3 newborns; *n* = 4 3-month-olds) or position bias (i.e., looking more that 85% of the time in one direction, *n* = 2 newborns, *n* = 2 3-month-olds). All newborns and infants were tested when they were awake and in an alert state. The protocol was carried out in accordance with the ethical standards of the Declaration of Helsinki (BMJ 1991; 302: 1194) and approved by the Ethics Committees of the San Gerardo Hospital and the University of Milan-Bicocca. Written informed consent was obtained from parents before the beginning of the study.

#### Stimuli

**Fig 1:**
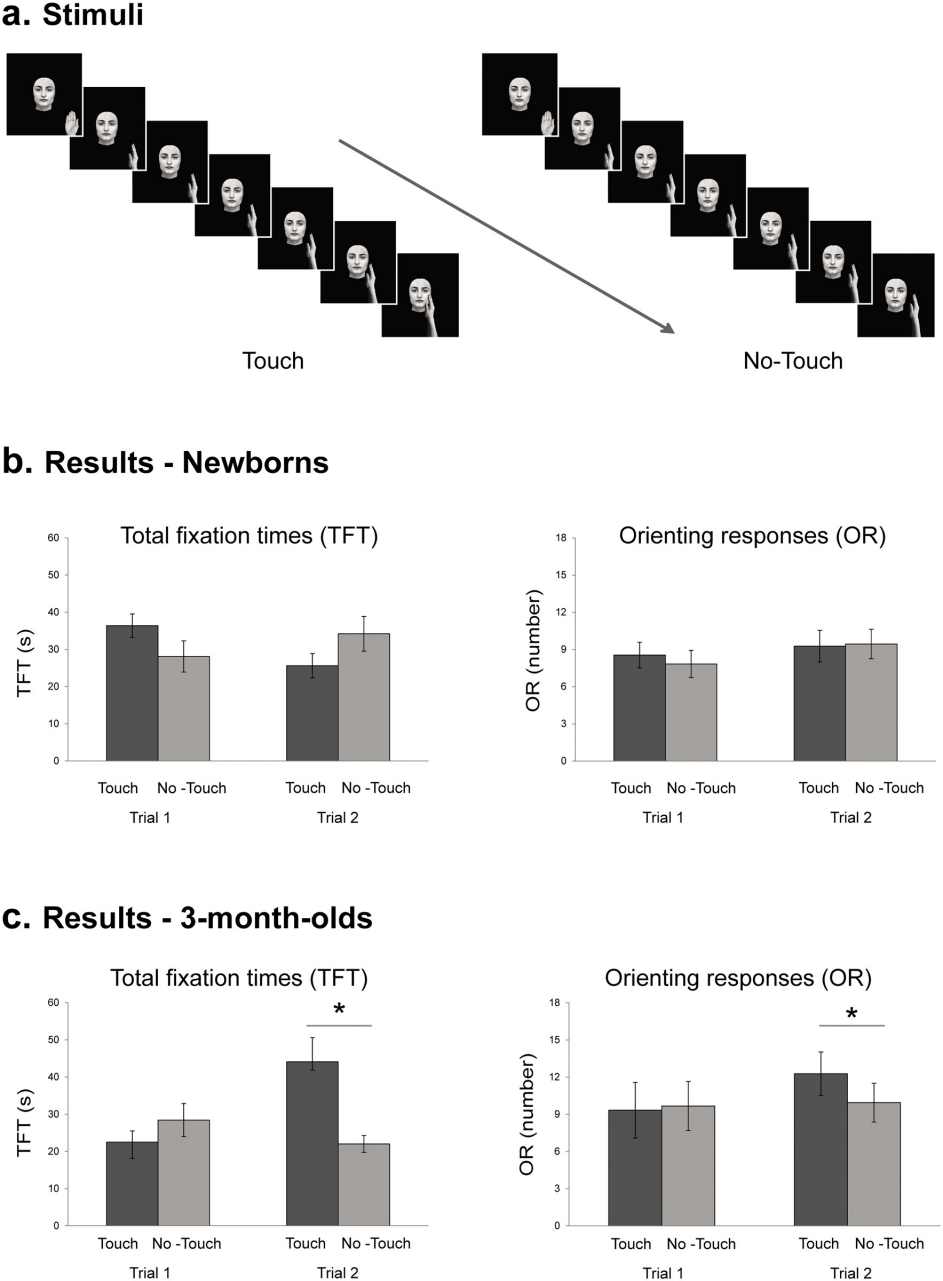
Stimuli and results of Experiment 1. (a) Frames composing the hand-to-face touching gesture (left) and the no-touching gesture (right) videos. Frames are presented in succession in the direction indicated by the arrow. The two stimuli were presented simultaneously on the screen (b) Newborns’ total looking times (left) and orienting responses (right) towards the touching and no-touching gesture during trial 1 and 2. (c) 3-month-old infants’ total looking times (left) and orienting responses (right) towards the touching and no-touching gesture during trial 1 and 2. Error bars refer to the standard errors of the mean. * = p < .05

Stimuli were two greyscale videos showing a hand moving towards a static young woman face on a black background. One video showed the hand reaching for the face and touching it on the cheek (touching gesture), while the other video showed the hand stopping at a distance of about 2.5 cm from the face (no-touching gesture). Each video comprised of seven frames. All frames depicted the face in the same frontal pose and central position within the image, whereas the hand changed position across the frames. The first two frames were the same for the touching and no-touching stimuli: Frame 1 depicted the hand in the lower corner of the image with the palm facing the observer; as the hand made a 90° rotation on the vertical axis, Frame 2 presented a sideway hand with the thumb in front and the other fingers aligned vertically. The following 5 frames showed the hand moving diagonally towards the static face, with an angle of 44° in the touching gesture and of 65° in the no-touching gesture (Fig 1). The amount of movement performed by the hand was the same on the vertical axis in the touching and no-touching stimuli, and differed between the stimuli on the horizontal axis (i.e., 0.5 cm). Both stimuli were presented simultaneously on the screen, and had the same duration (Video A in S1 File): each of the seven frames lasted 571 ms, for a total duration of 4 s. Luminance, contrast, and hue, as well as saturation, were kept constant between frames and stimuli. The videos of touching and no-touching gestures were presented bilaterally and played continuously, in a loop. The dimension of the hand at a distance of 40 cm from the screen ranged between 6.4° and 7.9° of visual angle in width, and between 14.2° and 24.7° of visual angle in height. The face was 14° wide and 21° high, and the distance between the faces depicted in the bilaterally presented videos was 39.2°. The, external contour of the eyes subtended a visual angle of 3.7° X 2°, and the eyes iris was 1 cm in diameter. The face stimulus was taken from our own database [38].

#### Procedure

Newborns were tested in a dedicated room at the hospital, whereas 3-month-olds were tested in a testing room at the University of Milan-Bicocca. An undergraduate student, blind to the aim of the study, sat with the newborn on the lap in front of the stimulus presentation monitor (27” screen size, 1920 X 1080 pixel resolution, 60 Hz) at a distance of about 30–40 cm. A video camera recording the newborns’ gaze direction was positioned above the monitor; the video camera sent a visual input to a laptop controlled by a second experimenter, who coded online newborns’ gaze and ran the experiment, which was designed with E-Prime 2 (Psychology Software Tools). Three-month-olds sat in an infant seat in front of the stimulus presentation monitor (24” screen size, 1600 X 1200 pixel resolution, 60 Hz), at a distance of about 40–50 cm. Both groups were tested using a preferential looking paradigm with an infant-control procedure. Stimulus presentation began as soon as the infant looked at a red flickering circle appearing in the center of the screen; after its disappearance, the two experimental trials were presented. In each trial, touch and no-touch stimuli were shown simultaneously and bilaterally on the screen. The left/right position of the stimuli in the first trial was counterbalanced across participants, and reversed between the first and the second trial. Between the two trials, the central circle re-appeared to attract the infants’ gaze. Each trial ended when the infant watched each stimulus at least once, and shifted their gaze away for more than 10 s. On average, newborns watched 16 (SD = 5.29) video sequences in the first trial and 15 (SD = 4.04) in the second trial, while 3-month-olds watched 13 (SD = 4.23) video sequences in the first trial and 16 (SD = 7.49) in the second one. Gaze direction and fixation times were coded online by the experimenter, who was blind to the left/right position of the stimuli on the screen; the number of orienting responses and total fixation times (i.e., sum of all fixations) on the stimuli were recorded as dependent variables [39]. The experimenter used right and left buttons of the mouse to code the corresponding newborns’ gaze direction (right, left or none); the duration of infants’ fixations corresponded to the duration of button pressing by the experimenter. Video-recordings of eye movements were coded offline for 50% of the infants by an observer, blind to the hypotheses of the study and the stimuli shown. Inter-coder agreement (Pearson correlation) was .91 for total fixation time and .82 for number of orientations for the newborns, and .97 and .99, respectively, for the 3-month-olds. The Intra-Class Correlation (ICC) coefficient was .94 for total fixation time and .87 for number of orientations for the newborns, and .98 and .99, respectively, for the 3-month-olds.

### Results

To determine whether newborns and 3-month-old infants were able to discriminate between touching and no-touching gestures, two separate repeated-measures Analyses of Variance (_rm_ANOVAs) were performed on total fixation times and number of orienting responses, with *trial* (first vs. second) and *gesture* (touching vs. no-touching) as within-subjects factors, and *age* (newborns vs. 3-month-olds) as the between-subjects factor. The _rm_ANOVA on total fixation times showed a significant *Trial x Gesture x Age* interaction, *F*_1,34_ = 9.12, *p* = .005, *η* ^*2*^ = .093, as well as a *Trial x Age* interaction, *F*_1,34_ = 5.20, *p* = .03, *η* ^*2*^ = .182. The 3-way interaction was explored through separate 2-way ANOVAs, one for each age group, with *trial* and *gesture* as within-subjects factors. For newborns, the analysis showed no significant main effects or interaction (all *p*s > .1) (Fig 1). For 3-month-olds, the analysis revealed a main effect of *trial*, *F*_1,17_ = 5.37, *p* = .033, *η* ^*2*^ = .046, *gesture*, *F*_1,17_ = 5.03, *p* = .038, *η* ^*2*^ = .053, and a significant *Trial x Gesture* interaction, *F*_1,17_ = 6.62, *p* = .020, *η* ^*2*^ = .160. The main effect of *gesture* showed that, overall, infants looked longer at the touching (*M* = 66.61, *SD* = 29.78) than at the no-touching gesture (*M* = 50.44, *SD* = 19.48). The preference for the touching gesture was apparent during the second trial, as revealed by the significant *Trial x Gesture* interaction. In fact, multiple post-hoc comparisons (by means of the Newman-Keuls test) showed that, during the second trial, infants looked longer at the touching gesture (*M* = 44.10, *SD* = 27.45) than at the no-touching gesture (*M* = 22.02, *SD* = 9.61), (p = .047, Cohen’s *d* = .78), whereas in the first trial they looked equally long at the touching (*M* = 22.51, *SD* = 12.65) and the no-touching gesture (*M* = 28.42, *SD* = 18.77) (p = .45, Cohen’s *d* = .22) (Fig 1). Looking times to the touching gesture were also significantly longer in the second trial (*M* = 44.10, *SD* = 27.45) than in the first trial (*M* = 22.51, *SD* = 12.65), (p = .031, Cohen’s *d* = .70). All the others comparisons failed to reach significance (all *p*s > .05).

The analyses on orienting responses confirmed and extended the results obtained for looking times. The 3-way _rm_ANOVA revealed a significant *Trial x Gesture x Age* interaction, *F*_1,34_ = 4.15, *p* = .049, *η* ^*2*^ = .005. Separate ANOVAs, with *trial and gesture* as within-subjects factors, were then performed for each age group. For newborns, the analysis did not reveal significant main effects or interactions (all *p*s > .2), whereas for 3-month-olds there was a main effect of *gesture*, *F*_1,17_ = 6.44, *p* = .021, *η* ^*2*^ = .031, as well as a significant *Trial x Gesture* interaction, *F*_1,17_ = 5.94, *p* = .026, *η* ^*2*^ = .056. The main effect of *gesture* showed that infants oriented their gaze more frequently towards the touching gesture (*M* = 21.61, *SD* = 16.23) as compared to the no-touching gesture (*M* = 19.61, *SD* = 14.43). However, multiple post-hoc comparisons (Newman-Keuls test) for the *Trial x Gesture* interaction showed that it was during the second trial that infants oriented their gaze more often to the touching gesture (*M* = 12.28, *SD* = 7.46), compared to the no-touching gesture (*M* = 9.94, *SD* = 6.65), (p = .008, Cohen’s *d* = .67). By contrast, in the first trial they oriented equally towards the touching (*M* = 9.33, *SD* = 9.52) and no-touching gesture (*M* = 9.66, *SD* = 8.39) (p = .67, Cohen’s *d* = .16) (Fig 1). Orienting responses in the second trial were also more frequently directed towards the touching gesture (*M* = 12.27, *SD* = 7.46) as compared to both the touching (*M* = 9.33, *SD* = 9.52) (p = .007, Cohen’s *d* = .55) and the no-touching gesture (*M* = 9.66, *SD* = 8.39) (p = .01, Cohen’s *d* = .61) of the first trial. All the others comparisons failed to reach significance (all *p*s > .6).

### Discussion

Three-month-olds, but not newborns, were able to differentiate between the two stimuli, as shown by longer looking times and more frequent orientations towards the touching than the no-touching hand-to-face gesture. A possible interpretation of the lack of discrimination in newborns may refer to the saliency of the face stimulus, which may trigger newborns’ attention, preventing them to differentiate the touching vs. no-touching gesture. Indeed, since the first hours of postnatal life faces are highly salient to infants, capturing their attention under a variety of conditions [40, 41, 42]. An alternative interpretation of the null result could be that the differences between the two gestures are not marked enough in our stimuli to be detected by newborns' immature visual system. The differences between the two gestures became more evident during the last frames of the videos and, thus, newborns may not have had enough time to detect them. With the aim of disentangling between these different interpretations, in Experiment 2 we investigated newborns’ ability to discriminate between touching/no-touching gestures when the tactile events are directed towards a body part other than the face.

## Experiment 2

Experiment 2 aims at exploring whether newborns are able to discriminate touching from no-touching gestures when presented with hand gestures directed towards a non-face body part, such as the hand. A further aim of Experiment 2 is to examine whether the social nature of the agent performing the gesture is critical in triggering newborns' visual sensitivity to touch. In order to address this issue, Experiment 2 introduced a new condition: a second group of newborns was presented with two dynamic images depicting an inanimate object approaching or touching a static hand.

### Method

#### Participants

The final sample included 34 healthy full-term newborns (13 girls; mean age: 46 h, range: 24–101, mean birth weight: 3410g, Apgar score: at least 8 after 5 minutes), recruited from the maternity unit of the San Gerardo Hospital in Monza. None of the newborns was reported to have a twin.

Newborns were assigned to two different experimental conditions: 17 newborns belonged to the social condition, 17 to the non-social condition. Eight additional newborns were tested but excluded from the final sample due to fussiness (*n* = 4) or position bias (i.e., looking more that 85% of the time in one direction) (*n* = 4). All newborns were tested when they were awake and in an alert state. The protocol was carried out in accordance with the ethical standards of the Declaration of Helsinki (BMJ 1991; 302: 1194) and approved by the Ethics Committees of the San Gerardo Hospital and the University of Milan-Bicocca. Written informed consent was obtained from parents before the beginning of the study.

#### Stimuli

**Fig 2:**
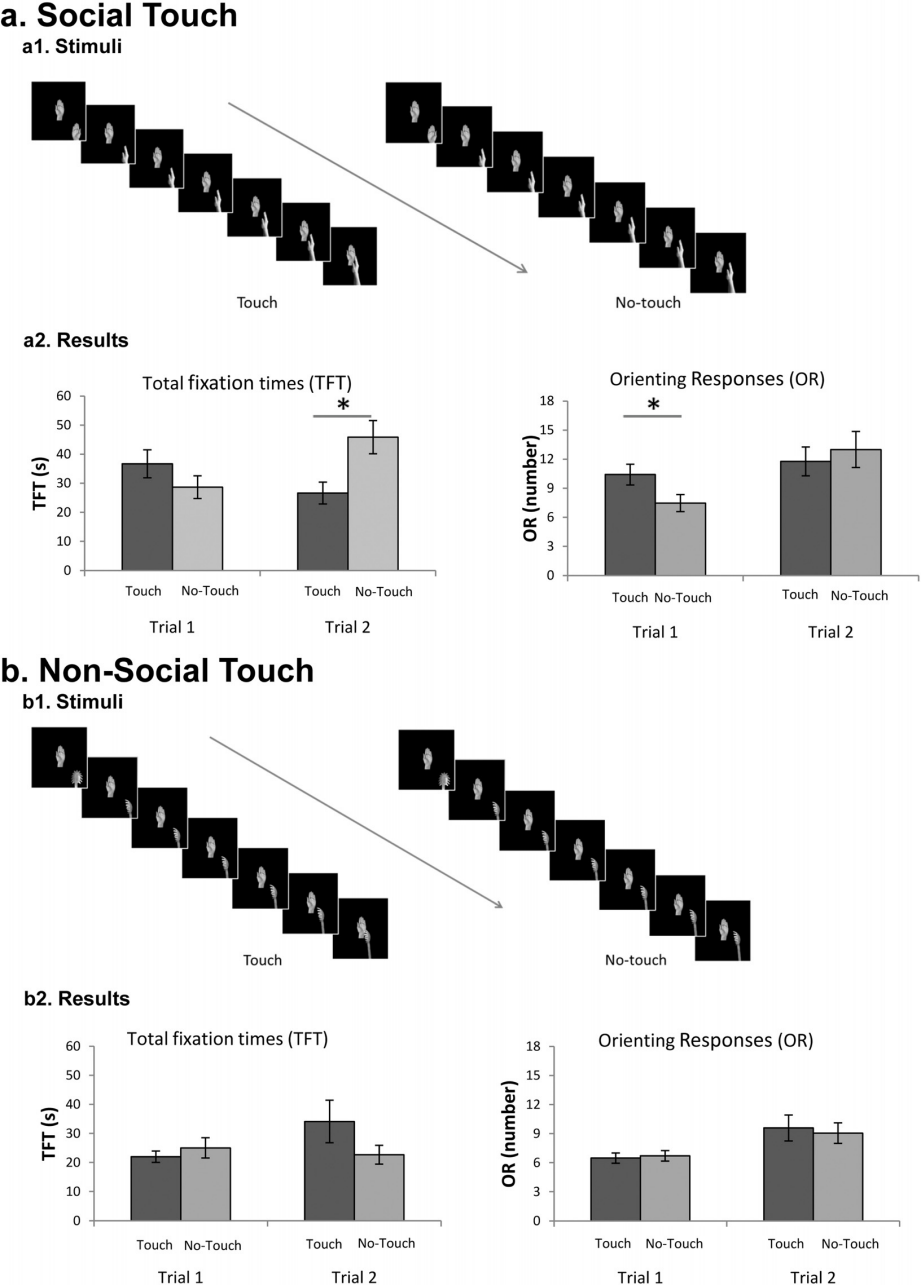
Stimuli and results of Experiment 2. (a) Stimuli and results of the social condition. (a1) Frames composing the hand-to-hand touching gesture (left) and the no-touching gesture (right) videos. Frames are presented in succession in the direction indicated by the arrow. The two stimuli were presented simultaneously on the screen (a2) Newborns’ total looking times (left) and orienting responses (right) towards the touching and no-touching gesture during trial 1 and 2 in the social condition. (b) Stimuli and results of the Non-social condition. (b1) Frames composing the object-to-hand touching gesture (left) and the no-touching gesture (right) videos. (b2) Newborns’ total looking times (left) and orienting responses (right) towards the touching and no-touching gesture during trial 1 and 2 in the Non-social condition. Error bars refer to the standard errors of the mean. * = p < .05

The technical features of the stimuli, as well as the experimental approach, were the same as in Experiment 1. Half of the newborns were presented with touching/no-touching gestures in which a hand moved towards a static hand (social condition) while the other half was presented with a spoon approaching a static hand (non-social condition) (Fig 2). Touching and no-touching stimuli were presented simultaneously on the screen (Videos B and C in S1 File). The dimension of the moving hand at a distance of 40 cm from the screen was the same as in Experiment 1, while the dimension of the spoon ranged between 4.3° and 9.3° of visual angle in width and between 14° and 24.7° of visual angle in height. The static hand was positioned with the palm facing the observer and was 7.9° wide and 17.8° high. The distance between the static hands depicted in the bilaterally presented videos was 39.2°. The moving spoon and the moving hand were positioned in each frame at the same distance from the static hand.

#### Procedure

The procedure was the same as in Experiment 1. Newborns watched an average of 14 (SD = 5.72) video sequences in the first trial and 14 (SD = 7.73) sequences in the second trial. The Intercoder agreement (Pearson correlation) on 50% of the participants was .82 for total fixation time and .86 for number of orientations. The ICC coefficient was .99 for both total fixation time and number of orientations.

### Results

The _rm_ANOVA on total fixation times with *trial* (first vs. second) and *gesture* (touching vs. no-touching) as within-subjects factors, and *condition* (social vs. non-social) as the between subject-factor showed a significant main effect of *condition*, *F*_1,32_ = 5.78, *p* = .022, *η* ^*2*^ = .055, and a significant *Trial x Gesture x Condition* interaction, *F*_1,32_ = 7.42, *p* = .010, *η* ^*2*^ = .08. The 3-way interaction was explored through separate 2-way ANOVA, with *trial* and *gesture* as within-subjects factors. For the non-social condition, the analysis showed no significant main effects or interaction (all *p*s > .1) (Fig 2). For the social condition, the analysis revealed a significant *Trial x Gesture* interaction, *F*_1,16_ = 6.71, *p* = .020, *η* ^*2*^ = .120. Based on the results obtained in Experiment 1, we explored this interaction with a set of planned comparisons, by means of t-test with the Bonferroni correction. During the first trial newborns looked equally longer at the touching (*M* = 36.68, *SD* = 19.83) and the no-touching gesture (*M* = 28.68, *SD* = 16.08), *t*_16_ = 1.299, *p* = .424, Cohen’s *d* = .315 (two-tailed), whereas in the second trial they looked significantly longer to the no-touching (*M* = 45.87, *SD* = 23.43) compared to the touching gesture (*M* = 26.64, *SD* = 15.41), *t*_16_ = 2.977, *p* = .018, Cohen’s *d* = .722 (two-tailed) (Fig 2).

The 3-way _rm_ANOVA performed on orienting responses revealed a significant main effect of *condition*, *F*_1,32_ = 4.32, *p* = .046, *η* ^*2*^ = .068, of *trial*, *F*_1,32_ = 17.58, *p* = .0002, *η* ^*2*^ = .088, and a significant *Trial* x *Gesture* x *Condition* interaction, *F*_1,32_ = 6.54, *p* = .015, *η* ^*2*^ = .014. Separate ANOVAs for each *condition* (Social, Non-social) with *trial* and *gesture* as within-subjects factors were then performed. For the non-social condition, the analysis revealed only a significant main effect of *trial*, *F*_1,16_ = 12.83, *p* = .002, *η* ^*2*^ = .215. Newborns oriented more frequently in the second trial (*M* = 18.65, *SD* = 8.35) than in the first one (*M* = 13.18, *SD* = 3.36), independently of the type of gesture. Differently, for the social condition there was a significant main effect of *trial*, *F*_1,16_ = 7.46, *p* = .015, *η* ^*2*^ = .204, as well as a significant *Trial* x *Gesture* interaction, *F*_1,16_ = 12.95, *p* = .002, *η* ^*2*^ = .075. Planned paired t-test (with Bonferroni correction) showed that in the first trial newborns oriented their gaze more frequently towards the touching (*M* = 10.41, *SD* = 4.40) than the no-touching gesture (*M* = 7.47, *SD* = 3.61), *t*_16_ = 3.178, *p* = .012, Cohen’s *d* = .77 (two-tailed), whereas in the second trial they oriented their gaze almost equally towards the touching gesture (*M* = 11.76, *SD* = 6.13) and the no-touching gesture (*M* = 13, *SD* = 7.63), *t*_16_ = 1.182, *p* = .508, Cohen’s *d* = .28 (two-tailed) (Fig 2).

Overall, these findings indicate that newborns are actually able to visually distinguish between touching and no-touching gestures involving two hands. Crucially, newborns do not manifest any preference when presented with a non-social touch, such as that provided by an inanimate object like a spoon.

## General Discussion

This study explored the developmental origins of the ability to visually recognize touching gestures involving human body parts (face, hand) and/or an object (spoon). In Experiment 1, only 3-month-olds, but not newborns, manifested a visual preference for a human hand-to-face touching gesture over a no-touching gesture. This indicates that 3-month-olds differentiated between a hand-to-face gesture that led to touch and a comparable gesture in which touch did not occur. Three-month-olds' discrimination between the hand-to-face touching and no-touching gesture was accompanied by a visual preference towards the touching gesture, which was evident for both fixation times and orienting responses.

In Experiment 2 we removed the potential interference effect generated by newborns’ sensitivity to faces by comparing newborns' gaze and looking behavior while watching touching and no-touching gestures directed towards a different human body part, namely a hand. Under this condition, 2-days-old newborns were able to differentiate between touching and no-touching gestures. Specifically, during the first trial newborns' attention was attracted by the touching gesture, as shown by newborns’ orienting responses. Then, in the second trial their attention was held for longer time by the no-touching hand-to-hand gesture, as testified by their total looking times. Crucially, newborns’ preference vanished when the agent of the gesture was an inanimate object, namely the spoon.

A possible interpretation of 3-month-olds’ spontaneous preference for the touching hand-to-face gesture is that gestures that comprise a tactile event are those that infants commonly experience during their daily interactions with others, and that provide them with both somatosensory and affective/communicative information. When observing a hand approaching a face, infants might expect the moving hand to fulfill its communicative and affective goal through touch, consequently making the hand-to-face touching gesture particularly salient and attractive for infants.

Three-month-olds’ preference for the touching gesture was apparent in the second trial. This might be due to the saliency of face, which may have captured infants' attention, to the point of masking the difference between the two hand-to-face gestures. Accordingly, infants appreciated the differences between the two hand gestures only during the second trial, when they shifted their attention from the face to the gesture. Future studies might confirm this interpretation by using an eye-tracker procedure to record infants’ scanning pattern on the stimuli.

Interestingly, newborns were able to distinguish touching from no-touching gestures when two hands were involved. Newborns’ ability to discriminate between these two gestures likely relies on the somatosensory-motor experience accumulated in the womb and in the first hours after birth. Such an experience might drive newborns' expectation that, when a hand is moving towards another hand, the approaching gesture will lead to contact, i.e. a touching event. In this vein, the switch in the direction of newborns' preference between the first and the second trial would imply a switch from a familiarity preference (i.e., for the familiar touching event) to a novelty preference (i.e., for the unexpected no-touching event). Irrespective of the factor driving the change in the direction of newborns' preference across trials, the crucial finding here is that newborns can discriminate touching versus no-touching hand gestures.

It is noteworthy that, in infant research, looking time is typically considered as a more sensitive measure of infants’ visual processing, than number of gaze orientations [43]. The direction of visual preference expressed by looking times in the current study was different for newborns and 3-month-olds infants, as the preference was towards the no-touching gesture for newborns in Experiment 2 and towards the touching gesture for 3-month-olds in Experiment 1. Unlike 3-month-olds, newborns have limited experience with interpersonal bodily interactions involving tactile contact and, thus, they might have also limited ability to decode the affective and communicative implications of touch. During prenatal life, fetuses’ tactile contacts are mainly related to the exploration of their own body and of the surrounding womb environment; within 3-months of postnatal life tactile experiences acquire an affective valence. The affective/communicative relevance that touch acquires through early interpersonal bodily experiences might explain the different visual behavior of newborns and 3-month-olds. On the other hand, there are indications that already during prenatal life fetuses have a natural predisposition to social interactions: when fetuses have to share the uterine environment with their co-twin, they touch and explore their twins’ body and these social contacts increase during the second semester of gestation [44]. This raises the question of the effect of such prenatal interpersonal contacts on newborns’ visual processing of touching/no touching gestures.

If newborns successfully discriminate between touching/no-touching body-related, potentially social, gestures thanks to their early somatosensory-motor experiences, their failure to show a preference in the presence of touches when the agent of the touch is an inanimate object may be attributed to their limited experience with gestures involving objects. However, it is important to note that the absence of a preference for either touching or no-touching object-to-hand gestures does not necessarily imply that newborns cannot discriminate them. They just might not have any expectation about the possible outcomes of a gesture that involves an object. The difference in newborns' visual behavior between the hand-to-hand and the object-to-hand condition of Experiment 2 supports the view that, shortly after birth, infants are tuned to human social gestures involving body-parts contact.

Taken together, the present evidence suggests that somatosensory-motor associations accumulated in the womb and in the first hours after birth may be sufficient to allow newborns to link executed with observed touching gestures. Moreover, infants’ spontaneous preferential responses to touching or no-touching gestures seem to be modulated by somatosensory-motor, visual and affective/communicative experiences. Finally, the fact that newborns’ early sensitivity to touch is specific to gestures with an higher social component, here a human body-to-body contact, suggests that the social nature of the gesture plays a key role in triggering newborns’ visual attention from the very early stages of postnatal life. In this regard, it is also noteworthy the evidence in human adults indicating that the human tactile mirror system, in particular some of its cortical regions, may be best tuned to represent human body-part interactions, than the contact between inanimate objects, or between human body-parts and objects [4, 13, 15], and it differentially responds to the sight of an intentional touch as compared to an accidental touch [9].

The sight of a touching, body-related, gesture provides infants with important information about the social world from very early in life, since touch implies a nonverbal communication of intentions and affect. Given fetuses’ organized and coordinated movements [22] and newborns’ early ability to detect synchrony between an observed and a felt body-related touch [26], we expected that from the earliest stages of postnatal life infants could visually discriminate between touching and no-touching gestures. Our results support this prediction, highlighting the importance of pre- and post-natal experience in the visual processing of touching gestures involving others' body.

## Supporting Information

S1 File Stimuli used in Experiment 1 and 2. (ZIP)
