# Correction: Seeing Touches Early in Life

**DOI:** 10.1371/journal.pone.0151294

**Published:** 2016-03-11

**Authors:** Margaret Addabbo, Elena Longhi, Nadia Bolognini, Irene Senna, Paolo Tagliabue, Viola Macchi Cassia, Chiara Turati

There are errors in the author affiliations. The affiliations should appear as shown here:

Margaret Addabbo^1^, Elena Longhi^1,2^, Nadia Bolognini^1,3^, Irene Senna^1,4^, Paolo Tagliabue^5^, Viola Macchi Cassia^1^, Chiara Turati^1^

**1** Department of Psychology and NeuroMi, Milan Center for Neuroscience, University of Milan-Bicocca, Piazza Ateneo Nuovo 1 (U6), 20126, Milano, Italy, **2** Research Department of Clinical, Educational and Health Psychology, University College London, London, United Kingdom, **3** Laboratory of Neuropsychology, Istituto Auxologico Italiano, Milano, Italy, **4** Cognitive Neuroscience Department and Cognitive Interaction Technology-Center of Excellence, Bielefeld University, Bielefeld, Germany, **5** Neonatology and Intensive Care Unit, MBBM Foundation, San Gerardo Hospital, 20900, Monza, Italy.
